# High levels of genetic diversity and population structure in an endemic and rare species: implications for conservation

**DOI:** 10.1093/aobpla/plw002

**Published:** 2016-01-14

**Authors:** Caroline Turchetto, Ana Lúcia A. Segatto, Geraldo Mäder, Daniele M. Rodrigues, Sandro L. Bonatto, Loreta B. Freitas

**Affiliations:** 1 Laboratory of Molecular Evolution, Department of Genetics, Universidade Federal do Rio Grande do Sul, PO Box 15053, Porto Alegre, 91501-970 Rio Grande do Sul, Brazil; 2 Laboratory of Genomics and Molecular Biology, Pontifícia Universidade Católica do Rio Grande do Sul, Av. Ipiranga 6681, Porto Alegre, 90619-900 Rio Grande do Sul, Brazil

**Keywords:** Conservation, genetic diversity, microendemic, microsatellites, plant evolution, plastid sequences

## Abstract

The analysis of genetic structure and variability of isolated species is of critical importance in evaluating whether stochastic or human-caused factors are affecting rare species. Low genetic diversity compromises the ability of populations to evolve and reduces their chances of survival under environmental changes. *Petunia secreta*, a rare and endemic species, is an annual and heliophilous herb that is bee-pollinated and easily recognizable by its purple and salverform corolla. It was described as a new species of the *Petunia* genus in 2005. Few individuals of *P. secreta* have been observed in nature and little is known about this species. All the natural populations of *P. secreta* that were found were studied using 15 microsatellite loci, two intergenic plastid sequences and morphological traits. Statistical analysis was performed to describe the genetic diversity of this rare species and the results compared with those of more widespread and frequent *Petunia* species from the same geographic area to understand whether factors associated with population size could affect rare species of this genus. The results showed that despite its rarity, *P. secreta* presented high genetic diversity that was equivalent to or even higher than that of widespread *Petunia* species. It was shown that this species is divided into two evolutionary lineages, and the genetic differentiation indices between them and other congeneric species presented different patterns. The major risk to *P. secreta* maintenance is its rarity, suggesting the necessity of a preservation programme and more biological and evolutionary studies that handle the two evolutionary lineages independently.

## Abstract (precis)

*Petunia secreta* is a rare and endemic species, that was found in two different landscapes, approximately 21 Km apart from each other. In this study we showed that *P. secreta* presented high genetic diversity that was equivalent to or even higher than that of widespread *Petunia* species. Two evolutionary lineages were found and they are correlated to the different landscapes where *P. secreta* grows: open areas in conglomerate sandstone towers at an elevation of approximately 300-400 m or along the road growing in an open vegetation flat area. Therefore the major risk to *P. secreta* maintenance is its rarity, suggesting the necessity of a preservation program.

## Introduction

Many species are rare or can be rare at some point during their existence. Various species maintain this rarity over the course of their existence; thus, one question is how can rare species maintain their population sizes when demographic challenges appear? Ecological and genetic explanations to rarity have been suggested. Rabinowitz (1981) proposed that rarity should be considered in three ways: geographic range (wide or narrow), habitat specificity (broad or restricted) and local abundance (somewhere large or everywhere small). Further, species could be rare in different senses, having many forms of rarity. Only one possible combination (wide range, broad habitat specificity and somewhere large local abundance) is classified as common species and all other combinations are different forms of rarity.

The hypothesis of niche breadth is one of several suggested mechanisms to explain species commonness and rarity; species that maintain populations in more varied environments may have a wide geographic distribution (Brown 1984; Slatyer *et al.* 2013). Niche and geographic distribution can be correlated with genetic variability. Species that present small population sizes and a restricted geographic range are expected to have reduced levels of genetic diversity (Hamrick and Godt 1989) because of natural selection or demographic phenomena believed to be more pronounced in these populations (Ellstrand and Elam 1993; Gibson *et al.* 2008). Insufficient variability would lead species to restrict their geographic range and be more vulnerable to extinction under novel selection pressure (Sheth and Angert 2014). However, whether and how the population would be affected by demographic processes, particularly genetic drift, depend on gene flow within and among populations (Choo *et al.* 2012), mating systems, pollen and seed dispersal, and effective population sizes (Kettle *et al.* 2007). Small populations might also experience higher levels of inbreeding, which might maximize the effect of genetic drift (Oleas *et al*. 2014).

Studies that analyse congeneric species account for phylogenetic relatedness (Felsenstein 1985) and are ideal for comparing genetic diversity of species with wide and restricted geographic distributions. Comparisons of different congeneric rare and widespread species could yield different patterns—in many cases, rare species maintain equivalent (i.e. Gitzendanner and Soltis 2000) or even higher (i.e. Ellis *et al.* 2006) levels of genetic diversity than do widespread congeners. However, in other situations, rare species have significantly lower levels of genetic diversity (i.e. Gibson *et al.* 2008). The lack of a constant pattern makes it important to study each rare species in order to critically evaluate its genetic variability and the drivers that shape it.

The genus *Petunia* (Solanaceae) is known worldwide through commercial garden petunias and 8 of its 14 species have a narrow geographic distribution. This genus is endemic to South America, showing a subtropical distribution ranging from 22° to 39°S (Stehmann *et al.* 2009). Several studies at the molecular level have been conducted involving *Petunia* wild species, and a common result of these analyses is the short genetic distances that are observed between taxa, indicating recent diversification of the genus (Ando *et al.* 2005; Kulcheski *et al.* 2006; Chen *et al.* 2007). As well, genetic variability in both plastid and nuclear markers is low (Kulcheski *et al.* 2006; Lorenz-Lemke *et al.* 2010; Reck-Kortmann *et al.* 2014).

**Figure 1.**
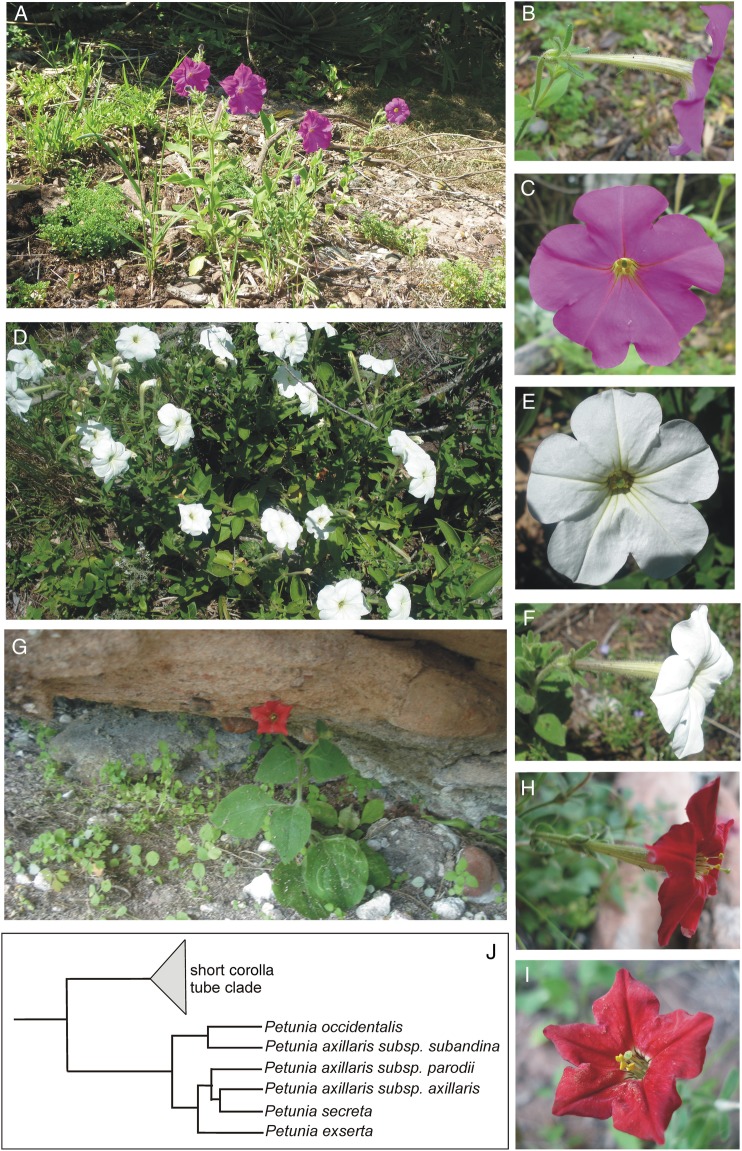
Studied species of *Petunia* from Serra do Sudeste highlighting flowers, habitats and phylogenetic relationship. (A–C) *Petunia secreta*, (D–F) *P. axillaris*, (G–I) *P. exserta* and (J) phylogenetic relationship among these taxa modified from Reck-Kortmann *et al*. (2014).

*Petunia secreta* is one rare, annual and heliophilous herb that is bee-pollinated and easily recognizable by its purple and salverform corolla (Fig. 1A–C). This taxon was collected for the first time in 1995 and described as a new species belonging to the *Petunia* genus in 2005 (Stehmann and Semir 2005). *Petunia secreta* is morphologically similar to *P. axillaris axillaris* and the corolla colour is the unique consistent trait distinguishing them (Stehmann and Semir 2005). Phylogenetic reconstructions suggest that *P. secreta* is the sister group of other taxa growing in the same geographic area [*P. axillaris* subsp. *axillaris* (hereafter *P. axillaris*; Fig. 1D–F and J) and *P. exserta* (Fig. 1G–J); Kulcheski *et al.* 2006] and shares with them a long corolla tube (Reck-Kortmann *et al.* 2014). Whereas *P. secreta* and *P. exserta* are distributed in a small geographical region, *P. axillaris* is widespread (Turchetto *et al.* 2014*a*, *b*).

**Figure 2.**
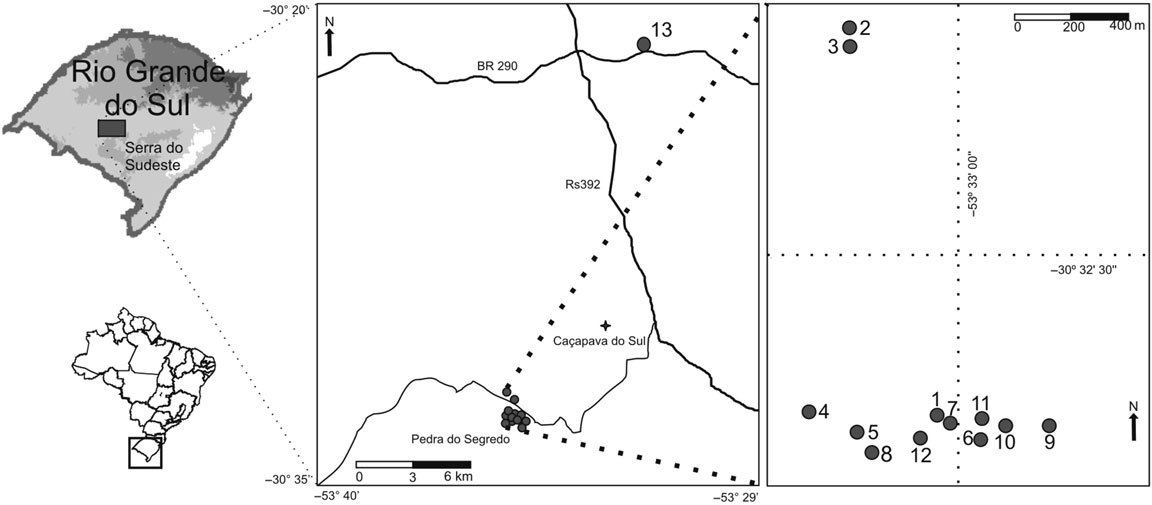
Simplified map showing the geographic distribution of collection sites of *P. secreta*. In detail, Pedra do Segredo locality and the distribution of 12 sampling sites.

*Petunia secreta* was originally portrayed as endemic to a small area called Pedra do Segredo municipality of Caçapava do Sul, Serra do Sudeste, southern Brazil (Fig. 2), growing in open areas in conglomerate sandstone towers at an elevation of ∼300–400 m. However, whereas *P. secreta* is restricted to Pedra do Segredo and its neighbourhood, *P. exserta* also has an endemic distribution and is found in a locality referred to as Guaritas in a rock formation, disjoint from Pedra do Segredo by ∼30 km (Lorenz-Lemke *et al.* 2006; Segatto *et al.* 2014*a*). There are no records of *P. secreta* in Guaritas region. *Petunia axillaris* species complex is widely distributed in temperate South America and the *axillaris* subspecies can be found growing in Guaritas in sympatry with *P. exserta* until ∼15 km from Pedra do Segredo but has never been observed within the geographic range of *P. secreta*. Since its discovery, few individuals of *P. secreta* have been seen in nature, and although it appears to be extremely rare, this species is not federally listed. Moreover, very little is known about the biology of *P. secreta*, and its genetic diversity has never been evaluated. One major question is centred on whether the few extant individuals in nature are sufficient to maintain adequate levels of genetic diversity.

In addition to their particular geographic range, these species present different floral syndromes and reproductive systems. Flowers of *P. secreta* are self-compatible (SC), non-fragrant and bee-pollinated (Stehmann and Semir 2005). The white flowers of *P. axillaris* are sweet and strongly fragrant after dusk (Gübitz *et al.* 2009) and mainly hawkmoth-pollinated (Ando *et al*. 1995; Venail *et al*. 2010; Klahre *et al*. 2011). However, *P. axillaris* individuals have more complex reproductive systems over the species geographic range. This subspecies was initially described as self-incompatible (Ando 1996), with observations of various SC individuals (Ando *et al*. 1998, 2001; Kokubun *et al.* 2006) at the edge of distribution. Recently, a study showed a mixed mating system for the *P. axillaris axillaris* with a high proportion of SC individuals in the sympatric area with *P. exserta* (Turchetto *et al*. 2015*a*). *Petunia exserta* is a SC species that exhibits red flowers, exserted reproductive organs and hummingbird-pollination (Watanabe *et al*. 2001; Lorenz-Lemke *et al*. 2006). With all *Petunia* species, the seeds fall close to the mother plant (Stehmann *et al.* 2009) and the plastid genome is maternally inherited (Derepas and Dulieu 1992). The goals of this study were to (i) describe the genetic diversity of the rare *P. secreta*; (ii) compare the genetic diversity of *P. secreta* with that of other rare (*P. exserta*) and widespread (*P. axillaris*) congeners and (iii) identify potential treats owing to rarity that may limit *P. secreta* persistence. The results of the work presented here have contributed to the knowledge on the relationships between the levels of genetic diversity and the geographic range size in clades of the *Petunia* genus, which contains rare and widespread plant species that share several morphological traits.

## Methods

### Sample collection

We searched for *P. secreta* individuals in the Pedra do Segredo locality and nearby beginning in 2006. Every year, only a few individuals were observed, and overall, 50 individuals from 12 occurrence sites were localized (Pop1–12; Table 1 and Fig. 2) all of which were in the Pedra do Segredo locality. However, during the spring of 2014, for the first time, new disjoint distributed individuals were found along the BR290 road and ∼21 km from Pedra do Segredo (Pop13; Fig. 2). At this new site, individuals grow in open vegetation flat area similar to that where *P. axillaris* individuals are seen. We collected randomly 23 of the >600 adult individuals from this new site. For every collection site, we obtained the geographical coordinates using a global positioning system unit and made exsiccates to deposit at the BHCB Herbarium (Universidade Federal de Minas Gerais, Belo Horizonte, Minas Gerais, Brazil; voucher numbers BHCB76025 and BHCB76027) and ICN Herbarium (Universidade Federal do Rio Grande do Sul, Porto Alegre, RS, Brazil; voucher numbers ICN181340, ICN181341, ICN181342, ICN181343, ICN181350 and ICN181352).

**Table 1.** *Petunia secreta* collection sites and plastid haplotype information per population.

Site	*N*	Haplotype	Sample site location	Geographic coordinates
Pop1	2	H1	Galpão de Pedra, Caçapava do Sul/RS	30°32′45.9″S/53°33′01.3″W
Pop2	1	H2	Pedra do Segredo, Caçapava do Sul/RS	30°32′08.2″S/53°33′10.5″W
Pop3	1	H3	Pedra do Segredo, Caçapava do Sul/RS	30°32′10.3″S/53°33′11.4″W
Pop4	1	H2	Galpão de Pedra, Caçapava do Sul/RS	30°32′45.7″S/53°33′15.0″W
Pop5	1	H4	Galpão de Pedra, Caçapava do Sul/RS	30°32′49.7″S/53°33′08.3″W
Pop6	1	H2	Galpão de Pedra, Caçapava do Sul/RS	30°32′47.5″S/53°32′57.5″W
Pop7	27	H1, H6, H7	Galpão de Pedra, Caçapava do Sul/RS	30°32′47.5″S/53°32′57.5″W
Pop8	1	H5	Galpão de Pedra, Caçapava do Sul/RS	30°32′48.8″S/53°33′08.8″W
Pop9	2	H2	Galpão de Pedra, Caçapava do Sul/RS	30°32′46.4″S/53°32′50.8″W
Pop10	1	H2	Galpão de Pedra, Caçapava do Sul/RS	30°32′46.3″S/53°32′55.3″W
Pop11	1	H2	Galpão de Pedra, Caçapava do Sul/RS	30°32′45.8″S/53°32′57.6″W
Pop12	11	H1, H8	Galpão de Pedra, Caçapava do Sul/RS	30°32′47.4″S/53°33′03.9″W
Pop13	15	H2, H9	BR290, km 330, Caçapava do Sul/RS	30°21′18.70″S/53°28′43.00″W

This work was conducted under permit MP 2.186/16 of the Brazilian Federal Government to access plant genetic information to develop evolutionary or taxonomic studies. No specific collection permits were required because *P. secreta* is not federally listed as endangered or protected and because no collection sites were based in protected areas.

### DNA extraction, amplification, sequencing and genotyping

Young leaves of 73 individuals of *P. secreta* were collected, such that injury and damage to the plants would be minimized, then dried in silica gel. We pulverized the leaves in liquid nitrogen for DNA extraction with cetyltrimethylammonium bromide as described by Roy *et al.* (1992). The quality and quantity of genomic DNA was evaluated by measuring the absorbance at 260 and 280 nm on a Nanodrop Spectrophotometer (NanoDrop 1000 spectrometer, Thermo Scientific Corp., USA).

The non-coding plastid *trnH-psbA* and *trnS-trnG* intergenic spacers of 65 individuals of *P. secreta* were amplified using universal primers as described by Hamilton (1999) and Sang *et al.* (1997), respectively, and following the amplification conditions according to Lorenz-Lemke *et al.* (2006). The polymerase chain reaction products were purified according to Dun and Blattner (1987) and sequenced in a MegaBACE 1000 (GE Healthcare Bio Sciences Corp., Piscataway, NY, USA) automatic sequencer according to the manufacturer's instructions and the DYEnamicET Terminator Sequencing Premix Kit (GE Healthcare). The sequences were deposited in GenBank **[see** **Supporting Information—Table S1****]**.

We also amplified 15 previously developed microsatellite markers [simple sequence repeat (SSR); Bossolini *et al.* 2011] that were scattered throughout the *Petunia* genome in 73 individuals of *P. secreta*. These markers were selected according to their location within the genome, their polymorphic index content (PIC) and their successful amplification in *P. secreta* samples. Polymerase chain reactions were conducted in a final volume of 10 µL, which contained ∼10 ng of genomic DNA as template following the protocol set forth by Turchetto *et al.* (2015*b*). The forward primers were FAM-, NED- or HEX-labelled. DNA amplicons were denatured and size-fractionated using capillary electrophoresis on a MegaBACE (GE Healthcare, USA) with a GeneTab 550 internal size ladder (GE Healthcare). The manufacturer's software was utilized to determine alleles.

### Genetic diversity and population structure based on plastid information

The 65 plastid sequences were manually aligned with GENEDOC software (Nicholas and Nicholas 1997). We used ARLEQUIN 3.5.1.2 (Excoffier and Lischer 2010) to estimate the basic descriptive molecular diversity statistics, such as haplotype and nucleotide diversities, and NETWORK 4.1.0.9 (http://www.fluxus-engineering.com/sharenet.htm) to estimate evolutionary relationships among the haplotypes. To evaluate the relationships of *P. secreta* with its congeneric species, a similar analysis was performed including plastid sequences obtained for *P. axillaris* and *P. exserta* generated by Turchetto *et al*. (2014*a*) and Segatto *et al*. (2014*a*), respectively.

Bayesian inference was performed as implemented in BEAST 1.6.1 (Drummond and Rambaut 2007) to estimate the phylogenetic tree using the different haplotypes of the three species. Two independent runs were employed consisting of 1 × 10^8^ Markov chain Monte Carlo (MCMC) iterations, sampling every 1000 generations under the HKY (Hasegawa, Kishino and Yano) nucleotide substitution model with four gamma categories in a Yule tree prior. Convergence was looked for in the stationary distribution by visually inspecting the posterior distribution of independent runs and ensuring that all parameters had effective sample sizes > 200 in TRACER 1.6 (Rambaut *et al.* 2013), removing the first 10 % iterations as a burn-in. A maximum clade credibility tree was obtained and the posterior probabilities for each node (Rannala and Yang 1996) using the TREEANNOTATOR software, part of the BEAST package. Finally, FIGTREE 1.4.0 (Rambaut 2008) was utilized to edit the phylogenetic tree.

The p-distance was also estimated in MEGA 6 (Tamura *et al.* 2013) to compare the mean intra- and interspecific genetic distances among *P. secreta*, *P. exserta* and *P. axillaris*. To detect evidence for deviation from a neutral equilibrium model of evolution in *P. secreta*, Tajima's *D* (Tajima 1989) and Fu's *F*_S_ (Fu 1997) neutrality tests were performed using the ARLEQUIN software. In these analyses, all haplotypes obtained for *P. secreta* were included and compared with those previously published for *P. axillaris* from Serra do Sudeste (Turchetto *et al.* 2014*a*) and *P. exserta* (Segatto *et al.* 2014*a*). In addition, changes in population size were verified over time for *P. secreta* by performing Bayesian skyline plot (BSP) analysis (Drummond *et al.* 2005) as implemented in BEAST package. For this analysis, a strict molecular clock model with a mean substitution rate of 2.8 × 10^−9^ per site per year (standard deviation 5.4 × 10^−11^) according to Lorenz-Lemke *et al*. (2010) and HKY nucleotide substitution model were used as priors. Markov chain Monte Carlo was performed for 100 000 000 steps, sampling every 10 000 steps. TRACER was employed to compute BSP and inspect for convergence. The program Alleles in Space 1.0 (Miller 2005) was made use of to associate the genetic and geographic distances between the two disjointed areas of *P. secreta* distribution according to Mantel's test (Mantel 1967). For this analysis, log-transformed geographic distance was utilized to compare the two *P. secreta* groups (Pop1–12 and Pop13).

### Genetic diversity and population structure based on SSR

FSTAT 2.9.3.2 software (Goudet 1995) was used to evaluate the summary statistics, such as the number of alleles per locus, gene diversity, allelic richness and inbreeding coefficient (*F*_IS_), for each locus. The frequencies of null alleles, PIC, levels of observed (*H*_O_) and expected (*H*_E_) heterozygosity and any significant deviations from the Hardy–Weinberg equilibrium (HWE) to evaluate the informativeness of the markers were estimated with CERVUS 3.0.3 software (Marshall *et al.* 1998; Kalinowski *et al.* 2007).

To investigate the genetic similarity among *P. secreta* individuals, discriminant analysis of principal components (DAPC; Jombart *et al.* 2010) was carried out as implemented in ADEGENET (Jombart 2008; R Development Core Team 2011). Discriminant analysis of principal components relies on data transformation from principal component analysis (PCA) prior to discriminant analysis, maximizing the separation between the groups. This analysis is not based on predefined population genetic models and makes no assumption about HWE or linkage disequilibrium. Whereas PCA and principal coordinates analysis focus on the entire genetic variation, DAPC searches for linear combinations of the alleles, enhancing the differences between groups while minimizing variation within clusters as measured by *F*-statistics. Discriminant analysis of principal components was performed with 13 groups set *a priori* representing the collection sites and also without prior information on the individual spatial origin. The number of clusters was assessed using *K*-means clustering and the optimal number of groups according to the Bayesian information criterion was selected. The number of PCs was set during the calculation process.

The genetic structure of *P. secreta* was examined using the STRUCTURE 2.3 software package (Pritchard *et al.* 2000) to determine the most likely number of independent genetic clusters (*K*). STRUCTURE was run without any prior information regarding sampling location. An admixture model was implemented using correlated allele frequencies (Falush *et al.* 2003) because gene flow is expected among natural populations. The best number of groups (*K*) was evaluated from 1 to 15, with 10 independent runs per *K* value. Each run was executed using 2.5 × 10^5^ burn-in periods, and 10^6^ MCMC repetitions after burn-in for population clustering were used. The optimal *K* value was identified from the maximum value of Δ*K* (Evanno *et al.* 2005) as implemented in STRUCTURE HARVESTER 0.6.93 (Earl and vonHoldt 2012). CLUMPP 1.1.2 (Jakobsson and Rosenberg 2007) was used to summarize the results of the optimal *K* value based on the pairwise similarity average of individual assignments across runs through Greedy's method and *G*′ statistics. DISTRUCT 1.1 (Rosenberg 2004) was employed to envisage the STRUCTURE results after processing with CLUMPP. One additional run of the STRUCTURE analysis took place to compare the 73 *P. secreta* with 25 *P. axillaris* from Serra do Sudeste and 24 *P. exserta* individuals, as previously studied by Turchetto *et al*. (2015*b*). Genetic differentiation among *P. secreta*, *P. axillaris* and *P. exserta* individuals was quantified, as well, through pairwise estimators of fixation index (*F*_ST_) via ARLEQUIN with 10 000 permutations to assess the significance, as well as the genetic differentiation between the two disjointed areas of *P. secreta* distribution (Pop1–12 and Pop13).

Mantel's test to compare the geographic (log-transformed) and genetic (*F*_ST_/1 − *F*_ST_) distances among populations was employed with statistical significance assessed via 10 000 permutations as implemented in ISOLDE as part of Genepop 4.2 package (Raymond and Rousset 1995).

A distance matrix was put together based on shared alleles among individuals **[see** **Supporting Information—Table S2****]** and collection sites **[see** **Supporting Information—Table S3****]** to depict the relationships among all of the *P. secreta* individuals using MSA 4.05 software (Dieringer and Schlötterer 2003). PHYLIP software (http://evolution.genetics.washington.edu/phylip.html) was made us of to construct an unweighted neighbour-joining (N-J) tree (Saitou and Nei 1987) based on the matrix of shared alleles, including 15 SSRs and 73 *P. secreta* individuals. In addition, genetic distance to compare *P. secreta*, *P. axillaris* and *P. exserta* individuals was acquired.

ARLEQUIN was also employed to estimate the Analysis of molecular variance (AMOVA; Excoffier *et al.* 1992) within *P. secreta*. In the variance analysis, the length of the amplified product of SSR markers of each accession as the value of microsatellite alleles was used.

To test for evidences of population reduction, BOTTLENECK 1.2.02 software (Piry *et al.* 1999) was utilized with 12 sites from Pedra do Segredo as one population and Pop13 as other. BOTTLENECK assumes one population that has undergone recent bottleneck presents more heterozygotes than expected under equilibrium. This was excess of heterozygotes was tested using a Wilcoxon signed-rank test (Cornuet and Luikart 1996) under a two-phase model (TPM) of microsatellite evolution (Di Rienzo *et al.* 1994) with a variance of 12 % (Piry *et al.* 1999) and different percentages of the stepwise mutation model in the TPM (70, 85 and 95 %) (Di Rienzo *et al*. 1998).

### Demographic parameters

Effective population size based on SSRs and plastid markers was estimated to the two disjointed areas of *P. secreta* occurrence (Pop1–12 from Pedra do Segredo and Pop13 from the road) using MIGRATE-N 3.6.11 software (Beerli 2009) with the Bayesian coalescent approach (Beerli and Felsenstein 2001; Beerli 2006). MIGRATE-N estimates Θ = 4Neμ for SSRs and Θ = 2Neμ for plastid markers, respectively (where Ne is the estimated population size and μ the mutation rate per loci per generation). To analyse the SSR markers, the Brownian motion model was used, with starting conditions based on *F*_ST_ and uniform prior distribution to estimate *θ* (range: 0–0.1). Our searches included one long chain with 10^7^ steps sampling, 10^4^ recorded genealogies and 10^5^ chains as burn-in. We ran four independent chains with distinct temperatures (1.0, 1.5, 3.0 and 10^5^) in a Markov Coupling (MCMCMC) procedure (Geyer and Thompson 1995) to ensure sampling over more genealogies. Estimates were computed per locus and summarized as weighted values over all loci. Estimates for plastid markers were performed using the sequence model summarizing the results of 10 replicates with 10^7^ steps sampling, 10^4^ recorded genealogies and 10^5^ chains as burn-in. The MCMCMC procedure (Geyer and Thompson 1995) was also used with distinct temperatures (1.0, 1.5, 3.0 and 6.0). *F*_ST_ was used to calculate starting conditions and a uniform prior distribution was used to estimate *θ* (range: 0–0.05). The same substitution rate used for the BSP analysis, 2.8 × 10^−9^ per site per year (Lorenz-Lemke *et al*. 2010), was used to estimate the number of individuals. We assessed stationary of the Markov chain checking the posterior distribution over all loci and running the programme several times with different starting points for both analyses.

### Morphological characterization

The morphology of flowers of 3 individuals from Pedra do Segredo and 12 individuals from the BR290 site was examined, one flower per individual. In the field, digital calipers measured three flower traits: the length of the corolla tube, the diameter of the corolla and the distance between the anthers of the longest and medium-length stamens **[see** **Supporting Information—Table S4****]**. Principal component analysis implemented in PAST software (Hammer *et al.* 2001) was able to determine whether the individual from the two disjoint locations could be distinguished by these three morphometric variables, as these morphological traits are important to discriminate subspecies in *P. axillaris* (Kokubun *et al.* 2006) and are associated with geographic distribution and genetic components (Turchetto *et al.* 2014*b*). The two principal components were plotted to evaluate the dispersion of the data.

### Conservation

The criteria adopted by the International Union for the Conservation of Nature (IUCN 2012) were used to identify the threat category of *P. secreta*. Conservation status was based mainly on the distribution area, as estimated with the help of GeoCAT—Geospatial Conservation Assessment Tool software (Bachman *et al.* 2011). The distribution area was estimated from the value of two parameters: the extent of occurrence and the area of occupancy. The conservation status followed the nomenclature of IUCN: Least Concern (LC), Not Threatened (NT), Vulnerable (VU), Endangered (EN) and Critically Endangered (CR).

## Results

### Genetic diversity and population structure based on plastid sequences

**Figure 3.**
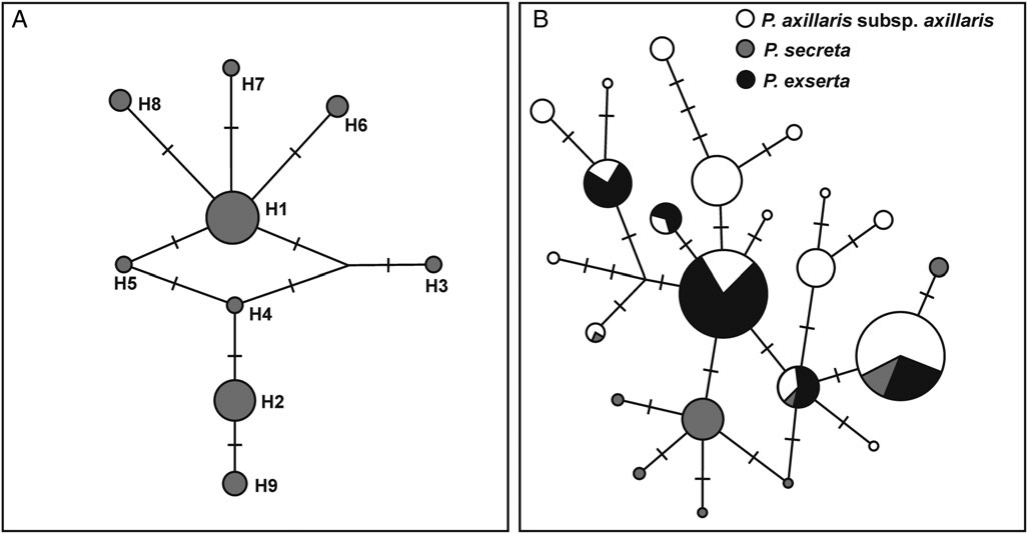
Evolutionary relationships of plastid haplotypes found in *P. secreta*. (A) *Petunia secreta* haplotypes. (B) *Petunia secreta*, *P. axillaris* and *P. exserta* haplotypes. The circles represent haplotypes, and the diameter is proportional to the frequency across 65 analysed individuals.

The plastids intergenic spacers, *trnH-psbA* and *trnS-trnG*, of 65 individuals of rare *P. secreta* were sequenced. Individuals were sampled in 13 different sites (Pop1–Pop13) (Table 1). The concatenated alignment was 1060 base pairs (bp) long (410 bp in *trnH-psbA* and 650 bp in *trnS-trnG*). These sequences possessed nine polymorphic sites (one transition and eight transversions), resulting in nine haplotypes (Fig. 3A). The evolutionary relationships among the nine haplotypes of *P. secreta* (Fig. 3A) did not exhibit a spatial correlation considering the different collection sites. Only at five collection sites, two or more individuals were found, and only in Pop7, Pop12 and Pop13, more than one haplotype was collected **[**Table 1; **see** **Supporting Information—Table S5****]**. The majority of individuals were of the H1 haplotype (35/65, from Pop1, Pop7 and Pop12), whereas H2 haplotype was found in 16/65 individuals from six sites (Pop2, Pop6, Pop9, Pop10, Pop11 and Pop13). The H9 haplotype was exclusive to individuals from Pop13 and at this collection site, only H2 was also found. Compared with *P. axillaris* and *P. exserta* haplotypes, six haplotypes were exclusive to *P. secreta* (H1, H5, H6, H7, H8 and H9), two were shared by the three species (H2 and H4) and one was shared strictly by *P. secreta* and *P. axillaris* individuals (H3) (Fig. 3B). One to three evolutionary steps separated these nine haplotypes of *P. secreta* (Fig. 3A). Haplotype diversity in *P. secreta* was *h* = 0.648 ± 0.051, and nucleotide diversity was *π* (%) = 0.17 ± 0.11 (Table 2).

**Table 2.** Genetic diversity of *P. secreta* based on plastid sequences compared with congeners. *N*, samples; the numbers in brackets represent private haplotype number; *P < 0.01. ^1^According to Turchetto *et al*. (2014*a*). ^2^According to Segatto *et al*. (2014*a*). ^3^Based on clustering analyses (I—Pop1–12; II—Pop13).

Species	*N*	*π* (SD), %	*h* (SD)	Haplotypes	Tajima's *D*	Fu's *F*_S_
*P. axillaris*^1^	359	0.21 (0.13)	0.770 (0.02)	23 [17]	−0.49	−2.60
*P. exserta*^2^	197	0.10 (0.10)	0.483 (0.036)	5 [0]	0.92	1.30
*P. secreta* I^3^	50	0.15 (0.10)	0.630 (0.076)	8 [5]	−0.81	−5.22*
*P. secreta* II^3^	15	0.05 (0.05)	0.514 (0.069)	2 [1]	1.38	1.25
*P. secreta* total	65	0.17 (0.11)	0.648 (0.051)	9 [6]	−0.16	−1.18

The Bayesian inference tree comparing the three species haplotypes **[see** **Supporting Information—Fig. S1****]** showed two fully supported clades, both including haplotypes of the three species. As expected, low support for the majority of the internal branches was observed.

**Figure 4.**
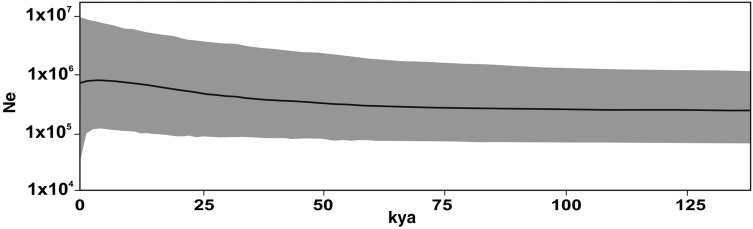
Bayesian skyline plot showing the fluctuations in effective population size (Ne) over time. The dark line indicates the median estimate and the grey area represents the 95 % highest posterior density intervals.

Based on plastid sequences, the interspecific genetic p-distance values were very low (0.002) among the three species, as was the intraspecific genetic distance in *P. secreta* (0.002) when considering all of the analysed individuals of this species. The neutrality Fu's *F*_S_ and Tajima's *D* tests (Table 2) to *P. secreta* were negative and not significant. The BSP (Fig. 4) suggested stability for *P. secreta* across time with a weak population growth signal beginning ∼60 kya, though this was not statistically significant given the size of the estimated confidence limits. The Mantel's test suggested a moderate association between genetic diversity and geographic distance based on plastid sequences considering the two groups of *P. secreta* (*r*^2^ = 0.5513; *P* < 0.001).

### Genetic diversity based on SSR

Seventy-three individuals of *P. secreta* were genotyped at 15 SSR loci and all markers were polymorphic. These markers yielded a total of 111 alleles, with an average of 7.4 alleles per locus (Table 3). The number of alleles per locus ranged from 2 (PM74) to 20 (PM177). The PIC values for these markers ranged from 0.06 (PM74) to 0.89 (PM177), with an average of 0.57, also demonstrating that these genetic markers were informative within this taxon (Table 3). Allele richness was, on average, 5.91 and ranged from to 1.81 (PM74) to 14.61 (PM177). The gene diversity was, on average, 0.63 and ranged from 0.06 (PM74) to 0.91 (PM177). The average *H*_O_ (0.24) was lower than the *H*_E_ (0.63) across all loci. Thirteen loci exhibited a significant departure from HWE expectations (*P* < 0.05) within the 73 investigated individuals. These deviations were characterized by high and positive *F*_IS_ values, except in relation to the PM183 locus (Table 3), indicating a deficit of heterozygotes. Heterozygote deficiency may be the result of biological factors, such as genetic drift or inbreeding, or indicators of null alleles. The frequency of null alleles was low (<1 %) across all loci.

**Table 3.** Genetic diversity indices based on the microsatellite profile in *P. secreta*. ^1^Including all individuals and loci; Chr, microsatellite location (chromosome number); SR, size range; *A*, number of alleles per locus; PIC, polymorphic index content; AR, allele richness; GD, gene diversity; *H*_O_, observed heterozygosity; *H*_E_, expected heterozygosity; *F*_IS_, inbreeding coefficient; NUL, frequency of null alleles. Bold values represent HWE deviation, significance after Bonferroni correction at *P* = 0.05.

Loci	Chr	SR	*A*	PIC	AR	GD	*H*_O_	*H*_E_	*F*_IS_	NUL (%)
PM188	I	100–145	11	0.83	9.38	0.85	**0.27**	0.85	0.68	0.52
PM195	I	184–214	7	0.57	5.12	0.65	**0.30**	0.64	0.54	0.36
PM101	I	237–270	7	0.59	6.41	0.63	**0.21**	0.62	0.67	0.50
PM21	II	89–128	3	0.38	2.56	0.50	**0.03**	0.49	0.94	0.88
PM88	II	142–170	7	0.64	5.77	0.70	**0.19**	0.70	0.72	0.56
PM183	III	102–174	11	0.65	7.42	0.70	0.69	0.70	0.02	0.01
PM191	III	164–170	3	0.44	2.98	0.53	**0.24**	0.53	0.55	0.38
PM8	IV	163–187	7	0.66	5.87	0.72	**0.24**	0.71	0.67	0.50
PM173	IV	157–187	5	0.56	4.47	0.62	**0.31**	0.62	0.51	0.34
PM74	IV	186–192	2	0.06	1.81	0.06	0.03	0.06	0.49	0.27
PM177	V	202–258	20	0.89	14.61	0.91	**0.32**	0.90	0.65	0.48
PM167	V	273–312	11	0.78	8.11	0.82	**0.33**	0.82	0.60	0.43
PM192	V	236–257	7	0.68	6.09	0.73	**0.27**	0.73	0.62	0.46
PM184	VII	90–108	7	0.45	5.06	0.51	**0.16**	0.51	0.69	0.53
PM206	IV	128–132	3	0.40	2.98	0.52	**0.06**	0.51	0.89	0.79
Average^1^			7.4	0.57	5.91	0.63	0.24	0.63	0.62	0.47
Pop1–12			6.2	0.51	5.19	0.55	**0.08**	0.55	0.56	0.40
Pop13			3.5	0.35	3.38	0.41	**0.19**	0.40	0.47	0.35

The genetic diversity estimates of *P. secreta*, as allele richness, *H*_O_, *H*_E_ and *F*_IS_ values were statistically compared with the same estimates obtained for *P. axillaris* (25 individuals from Guaritas) and *P. exserta* (23 individuals also collected in Guaritas) (see Table 1 in Turchetto *et al*. 2015*b* for details of locality of sample: *P. axillaris* Pop4–7 and 9–11, *P. exserta* Pop26–32 and 34). The results were as follows: allele richness (*P. secreta* > *P. exserta*, *P. secreta* = *P. axillaris* and *P. axillaris* > *P. exserta*), *H*_O_ (*P. secreta* = *P. exserta*, *P. secreta* < *P. axillaris* and *P. axillaris* > *P. exserta*), *H*_E_ (similar for all comparisons) and *F*_IS_ values (*P. secreta* = *P. exserta*, *P. secreta* > *P. axillaris* and *P. axillaris* < *P. exserta*). The allele richness of a sample is affected by the size of a sample (large samples are expected to have more alleles than small samples) and can produce unbiased estimates. Because of this, we also estimated the allele richness in HP-RARE 1.0 software (Kalinowski 2005), on which the statistical technique of rarefaction compensates for sampling disparity. These results were similar to those described above (data not shown).

### Population structure based on SSR

**Figure 5.**
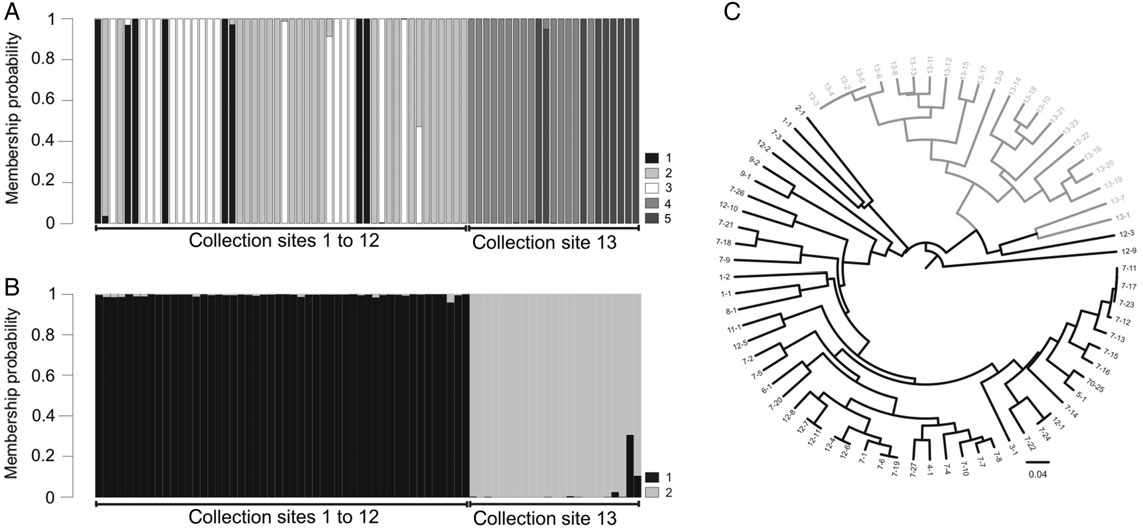
Population structure and evolutionary relationships of *P. secreta* individuals based on 15 microsatellite loci as observed through clustering analyses in the DAPC scatter plot (A) and STRUCTURE (B). Different colours indicate groups (DAPC, *K* = 5; STRUCTURE, *K* = 2), and vertical lines correspond to individuals. Neighbour-joining tree (C) obtained from a distance matrix based on shared alleles among individuals and collection sites (Pop13 individuals are presented in grey). Individual numbers according to describe in **Supporting Information—Table S1** **[see** **Supporting Information—Table S1****]**.

*Petunia secreta* individuals formed five genetically homogeneous groups according to DAPC analysis (Fig. 5A) with three of them grouping individuals from Pedra do Segredo (Pop1–12), whereas two clusters encompassed the individuals from Pop13. The Bayesian clustering analysis as implemented with the admixture model in STRUCTURE (Fig. 5B) revealed two genetic components to 73 *P. secreta* individuals according to the Evanno *et al.* (2005) method (best *K* = 2), splitting the sample into two major groups relative to the collection location (Pop1–12 or Pop13). The same results were obtained when prior information about the collection points was included in the STRUCTURE analysis (data not shown). Only a few individuals in each group showed some degree of admixture. According to Mantel's test, the genetic differentiation between the two groups in *P. secreta* was weekly associated with geographic distance (*r*^2^ = 0.1255; *P* < 0.001).

**Figure 6.**
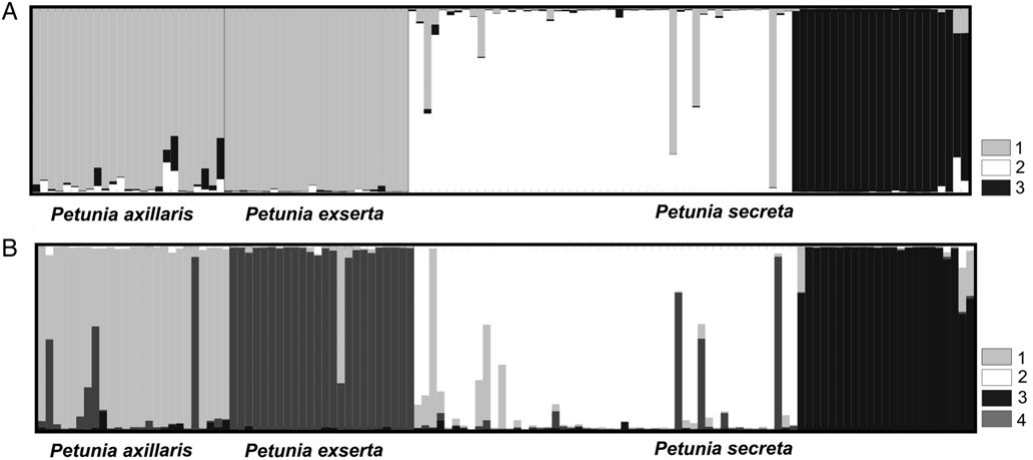
Evolutionary relationships among different *Petunia* taxa as inferred from microsatellite loci according to the STRUCTURE clustering analysis best *K* = 3 (A) and *K* = 4 (B). Each colour represents a genetic component and the vertical lines represent individuals. The *P. axillaris* and *P. exserta* genetic profiles were obtained from Turchetto *et al.* (2015).

To identify the evolutionary relationships between *P. secreta* and other *Petunia* species based on genetic components according to SSR alleles, a STRUCTURE analysis was conducted based on 14 SSR loci, as described by Turchetto *et al*. (2015*b*) for *P. axillaris* and *P. exserta*, and the 73 *P. secreta* individuals. *K* values ranging from 1 to 15 were tested. The best number of groups was three (*K* = 3), separating *P. secreta* individuals into two groups (all collection sites from Pedra do Segredo and Pop13) and the third group corresponding to *P. axillaris* subsp. *axillaris* and *P. exserta* individuals (Fig. 6A). When *K* = 4, *P. axillaris* and *P. exserta* became separated (Fig. 6B), and two groups followed as observed for *P. secreta*.

Based on *F*-statistic, larger genetic distance was observed between *P. secreta* and *P. exserta* (*F*_ST_ = 0.195; *P* < 0.001) versus *P. secreta* and *P. axillaris* (*F*_ST_ = 0.080; *P* < 0.001) and *P. axillaris* and *P. exserta* (*F*_ST_ = 0.113; *P* < 0.001). The highest genetic differentiation was seen when comparing the two disjoint sites for *P. secreta* (Pop1–12 and Pop13; *F*_ST_ = 0.358; *P* < 0.001).

The genetic distance based on shared alleles among the collection sites of *P. secreta* **[see** **Supporting Information—Table S3****]** was the lowest between Pop7 and Pop12 (0.27) and the highest between Pop2 and Pop9 (0.83). Comparing *P. secreta* with the other *Petunia* species based on the 14 in-common analysed SSR **[see** **Supporting Information—Table S6****]**, *P. secreta* was closer to *P. axillaris* (0.42) than to *P. exserta* (0.50). Accounting for the samples from Pedra do Segredo as one group and Pop13 as another based on their geographic distances **[see** **Supporting Information—Table S7****]**, the genetic distance between these groups (0.63) was higher than that observed between *P. axillaris* subsp. *axillaris* and *P. exserta* (0.42), and Pop13 was more different from these species than the remaining populations were.

The N-J tree (Fig. 5C) presented a similar topology to that of the STRUCTURE results and was divided into two main groups. In this analysis, only one individual from Pop12 was closer to the group that was formed by Pop13 individuals, and no individuals from Pop13 were outside this group. Individuals from the same site from Pedra do Segredo did not form preferential groupings.

An AMOVA revealed that higher molecular variance is equally distributed among populations (34 %), within individuals (33 %) and among individuals within populations (34 %). The *F*-statistic over all loci was significant at the level of 0.001 (*F*_ST_ = 0.34, *F*_IS_ = 0.51 and *F*_IT_ = 0.67). When just the 12 sites from the Pedra do Segredo locality were considered, the most variation was found among individuals within populations (51 %) than among populations (23 %) or within individuals (26 %). The *F*-statistic over all loci was significant at *F*_ST_ = 0.229 (*P* < 0.001), whereas *F*_IS_ (0.665) and *F*_IT_ (0.742) were not significant (*P* > 0.01).

No excess of heterozygotes was detected with BOTTLENECK for the collection sites Pedra do Segredo (Pop1–12) and Pop13 (lower *P* value in Wilcoxon test; *P* = 0.92 and *P* = 0.88, respectively), indicating that the populations did not undergo a founder effect. However, this result should be interpreted with caution as many loci were not in HWE.

### Effective population sizes

Estimated Θ values based on SSRs markers were similar between Pop1–12 (0.099; with 95 % confidence intervals of 0.097–0.1) and Pop13 (0.097; with 95 % confidence intervals of 0.095–0.1). The effective population size based on plastid markers, in number of individuals, using the substitution rate estimated for *Petunia* (Lorenz-Lemke *et al*. 2010) was 745 individuals for Pop1–12 (Θ = 0.00172) and 485 for Pop13 (Θ = 0.00112).

### Morphological variability

Principal component analysis showed that the percentage of variance was 84.5 for the first component and 9.5 % for the second component. The 95 % distribution included all of the individuals **[see** **Supporting Information—Fig. S2****]** and confirmed that individuals from all of the collection sites corresponded to the morphological description of *P. secreta* previously put forth by Stehmann and Semir (2005). The corolla colour was also in agreement with the original description.

### Conservation

According to IUCN (2012) criteria, the conservation status of *P. secreta* was classified as critically endangered [CR: B1ab(ii, iii, iv)] because of the species' extremely limited extent of occurrence (81 km^2^). This species inhabits an area with high fragmentation, reducing the area of occupancy (20 km^2^) and, consequently, decreasing habitat quality. Moreover, *P. secreta* has been found in just two different locations.

## Discussion

Conservation biologists are interested in knowing whether there are generalities that can be made with regard to rare species, such as whether these species typically exhibit reduced genetic diversity or restricted gene flow between populations, as predicted by population genetic theory when the populations are small and isolated, with profound evolutionary and ecological consequences. Ecological and genetic explanations to rarity have been suggested. Both genetic variation in ecologically relevant traits and differences in selection pressures, in addition to neutral forces, such as the significant impact of genetic drift and inbreeding in small populations of rare plants, can drive the geographic distribution of a species (Sheth and Angert 2014). An important question in conservation genetics is how does genetic variability compare among rare plants and their widespread related species (Furches *et al.* 2013).

The IUCN criteria have been used to assess conservation status at a species level. Here, the status of *P. secreta* is classified as Critically Endangered. Additionally, as proposed by Rabinowitz (1981), *P. secreta* could also be considered as a rare species exhibiting geographic range and small populations, but it is broad because individuals grow in two different habitats. There are biological, ecological and evolutionary mechanisms that allow many rare plant species to persist. Species currently rare may have become so in recent history (Bekker and Kwak 2005). Moreover, Schwartz and Simberloff (2001) observed a trend for rare species to be included into species-rich clades, suggesting that speciation and extinction might be linked. Knapp (2011) made note of the fact that, for example in Cape flora region, threatened species are more common in clades that are young and diversifying quickly, signifying that risk is conserved in lineages but differs wildly at the tips of those lineages. These differences can be associated with the mode of speciation. For example, plants that speciate via small isolated populations at the edges of a large species range present rapid diversification and will have a larger numbers of threatened species. Moreover, plants diversifying through peripatry could have large variations in threats among the tips of phylogenetic trees (Davies *et al*. 2011).

With only 13 known collection sites (Fig. 2) scattered over a small area, *P. secreta* is a perfect example of a rare plant that has a restricted geographical range and small local populations. Given its ecological characteristics and despite the general expectation of reduced genetic variation in a rare species, *P. secreta* possesses high genetic variation, as denoted by the extensive microsatellite polymorphism and plastid haplotype numbers, and no evidence of having gone through a recent founder effect. The results ultimately show that the species persisted over time (Fig. 4) and was able to grow in varied habitats, occurring on the rocks in Pedra do Segredo and along the road in another kind of soil. Moreover, plants from Pop13 are higher and display bigger leaves than those collected from Pedra do Segredo probably because of soil richness and moisture.

Compared with other *Petunia* species, *P. secreta* displays one of the highest diversity values. Based on the same microsatellite set, Turchetto *et al*. (2015*b*) found 90 alleles with an average of 6 alleles per locus and an allele richness average of 6.3 in *P. axillaris*, and 68 alleles with an average of 4.5 per locus and an allele richness average of 4.8 in *P. exserta*. Comparing three species of the *P. integrifolia* complex based on seven microsatellite loci, Segatto *et al*. (2014*b*) observed lower values, especially in relation to the allele richness (3.73 in *P. inflata*, 3.29 in *P. integrifolia* subsp. *integrifolia* and 4.39 in *P. interior*). The geographic range of these *P. integrifolia* complex species and the number of individuals per occurrence site are much larger than those observed for *P. secreta* (except Pop13). Obtained Θ values based on nuclear markers are bigger than those obtained for *P. exserta* (Θ = 0.057) and smaller than that estimated for *P. axillaris* (Θ = 0.43) by Segatto *et al.* (2014*a*). Considering plastid markers, the effective population size of *P. secreta* is comparable with that of *P. axillaris* (Θ = 0.0025).

Plants with restricted distributions are expected to exhibit low genetic diversity at the species level (Cole 2003). In addition, fragmentation into isolated and discrete demes composed of relatively few individuals contributes to a depletion of overall genetic diversity (Ellstrand and Elam 1993). The genetic diversity found in *P. secreta* is comparable with that in other plant species associated with narrow geographic distribution and small populations (Furches *et al.* 2013), or even those much more widespread (Koopman and Carstens 2010).

The extension of the geographic range alone either should not be used to reliably predict genetic dynamics (Premoli *et al.* 2001) or constitutes a strong enough reason to guide conservation actions. Indeed, there are several other factors that influence the levels of diversity within and among populations, such as ecological and biogeographical factors and mating systems (Wang *et al.* 2004; Pérez de Paz and Caujapé-Castells 2013; Coppi *et al.* 2014). Additionally, a number of causes of rarity have been proposed, like ecological equivalency, frequency-dependent selection and narrow niche requirements, and these factors play an important role in the abundance of species (Markham 2014).

As demonstrated here, genetic variability and population structure descriptors suggest ancient variability and a stable founder population to *P. secreta*. Populations of *P. secreta* from Pedra do Segredo (Pop1–12) presented a higher partitioned variation among individuals (51 %), suggestive of this species being part of a single panmictic group. Moreover, all of the analyses indicate that Pedra do Segredo and Pop13 constitute different units. Consequently, *P. secreta* is a narrowly distributed species that is primarily distributed into two large populations genetically differentiated and occurring in a fragmented area, ∼20 km from each other.

The environmental conditions are different between the major localities where *P. secreta* was found, especially the soil substrate and associated vegetation. Whereas plants in Pedra do Segredo grow directly on the rock in shallow soil, close to cacti and other xerophyte plants, the plants along the road grow in deep and rich soil and are surrounded by grass in a completely modified environment. Though the floral traits did not have significant differences between individuals and the two groups (Pop1–12 and Pop13) presenting morphological characteristics, as proposed in the *P. secreta* description, the genetic data of each population correspond to different evolutionary lineages both in the plastid haplotypes and in the microsatellites (see Figs 3 and 5). The results indicate a lack of connectivity by pollination or seed dispersion between Pop13 and the Pedra do Segredo group. Besides, Pop 13 has an effective population size about half of the Pop1–12 when plastid markers are considered.

Genetic information may be important for conserving and recovering rare species. However, these rare species may not always be genetically depleted (Edwards *et al.* 2014). Though high genetic diversity is not a common occurrence for rare species, Gitzendanner and Soltis (2000) demonstrated that endangered species occasionally exhibit levels of diversity as high as or higher than that of a widespread congener. *Petunia secreta* presents indices of genetic variability that are similar to *P. exserta* that is also endemic to a small area and lower than *P. axillaris*, which is distributed in a larger range. The three species possess different floral syndromes and reproductive systems, possibly influencing the different levels of diversity seen. Although *P. exserta* and *P. secreta* are SC, higher allelic richness was observed within *P. secreta*. As a consequence of relying on just a few pollinator species for sexual reproduction, low density or narrow geographical range of the pollinator can contribute to rarity of the plants (Phillips *et al*. 2014). Despite the high level of genetic diversity, a decrease in heterozygosity was evident (13 loci deviated from HWE, and no significant null alleles were observed), suggesting a significant amount of inbreeding in *P. secreta* as a consequence of self-fertilization and/or preferential mating among relatives. A spatial genetic structure was observed across collection sites and groups formed in Bayesian inference tree, clustering and DAPC analyses, suggesting the presence of two evolutionary lineages in *P. secreta* with some gene flow between neighbouring sites. The individuals from Pop13 were less variable than the individuals from Pedra do Segredo (Pop1–12) and had only a subset of the entire species' set of SSR alleles. This difference could be from a founder effect or genetic drift. The higher differentiation between Pop13 and other *P. secreta* populations compared with that between *P. axillaris* and *P. exserta*, as evidenced by STRUCTURE (Fig. 5), could be a consequence of the assumptions within this analysis.

One major risk to *P. secreta* maintenance is that it is composed of only two effective population or evolutionary lineages (Pop1–12 from Pedra do Segredo and Pop13) because no other natural populations were found despite the exhaustive search for this species along the area for ca. 10 years. It is suggested that in order to protect against loss of genetic variability and extinction of species, seeds are collected from as many individuals as possible and stored in seed banks and living collections, thus promoting regular genetic monitoring to restore eventual stochastic losses. Moreover, *P. secreta* needs to be federally listed as a threatened species, and the two different groups should be treated at least as management units.

Further experiments are necessary to gather knowledge on the mating system and understand the complete evolutionary history of *P. secreta*.

## Conclusions

The results from this work demonstrate that even within the same category of rarity, different species might exhibit a range of spatial and temporal characteristics, of which the outcome is differential genetic patterns. Here, it is shown that although *P. secreta* is intrinsically rare, this rarity is not a feature that negatively affects genetic diversity; the fragmented distribution preventing gene flow between major populations is what puts the species' persistence at risk.

## Sources of Funding

This work was supported by the Conselho Nacional de Desenvolvimento Científico e Tecnológico (CNPq), Coordenação de Aperfeiçoamento de Pessoal de Nível Superior (CAPES) and the Programa de Pós-Graduação em Genética e Biologia Molecular da Universidade Federal do Rio Grande do Sul (PPGBM-UFRGS).

## Contributions by the Authors

C.T. and L.B.F. planned, designed and led the project; C.T., A.L.A.S., G.M. and D.M.R. conducted the experiments; C.T., A.L.A.S., G.M. and S.L.B. ran the analyses; C.T., A.L.A.S., G.M. and L.B.F. wrote most of the text; L.B.F. and S.L.B. provided reagents and equipment to develop the experiments. All authors contributed in the preparation of the study and have commented on and approved the final manuscript.

## Conflict of Interest Statement

None declared.

## Supporting Information

The following additional information is available in the online version of this article –

**Table S1.** GenBank accession numbers.

**Table S2.** Genetic distance based on SSR alleles.

**Table S3.** Genetic distance based on microsatellite per collection site.

**Table S4.** *Petunia secreta* flower measurements.

**Table S5.** Plastid haplotypes per individual per collection site.

**Table S6.** Genetic distance based on SSR among *Petunia* species.

**Table S7.** Genetic distance based on SSR among *Petunia* species separating *P. secreta* into two subgroups.

**Figure S1****.** Evolutionary relationships among plastid haplotypes.

**Figure S2****.** Principal component analysis based on morphology.

Additional Information

## References

[PLW002C1] Ando T 1996 Distribution of *Petunia axillaris* (Solanaceae) and its new subspecies in Argentina and Bolivia. Acta Phytotaxonomica et Geobotanica 47:19–30.

[PLW002C2] Ando T, Tida S, Kokubun H, Ueda Y, Marchesi E 1995 Distribution of *Petunia axillaris* sensu lato in Uruguay as revealed by discriminant analysis of the live plants. Journal of the Japanese Society for Horticultural Science 64:381–391. 10.2503/jjshs.64.381

[PLW002C3] Ando T, Tsukamoto T, Akiba N, Kokubun H, Watanabe H, Ueda Y, Marchesi E 1998 Differentiation in the degree of self-incompatibility in *Petunia axillaris* (Solanaceae) occurring in Uruguay. Acta Phytotaxonomica et Geobotanica 49:37–47.

[PLW002C4] Ando T, Nomura M, Tsukahara J, Watanabe H, Kokubun H, Tsukamoto T, Hashimoto G, Marchesi E, Kitching J 2001 Reproductive isolation in a native population of *Petunia* sensu Jussieu (Solanaceae). Annals of Botany 88:403–413. 10.1006/anbo.2001.1485

[PLW002C5] Ando T, Kokubun H, Watanabe H, Tanaka N, Yukawa T, Hashimoto G, Marchesi E, Suaréz E, Basualdo IL 2005 Phylogenetic analysis of *Petunia* sensu Jussieu (Solanaceae) using chloroplast DNA RFLP. Annals of Botany 96:289–297. 10.1093/aob/mci177 15944177 PMC4246877

[PLW002C6] Bachman S, Moat J, Hill AW, de la Torre J, Scott B 2011 Supporting Red List threat assessments with GeoCAT: geospatial conservation assessment tool. ZooKeys 150:117–126. 10.3897/zookeys.150.2109 PMC3234434 22207809

[PLW002C7] Beerli P 2006 Comparison of Bayesian and maximum-likelihood inference of population genetic parameters. Bioinformatics 22:341–345. 10.1093/bioinformatics/bti803 16317072

[PLW002C8] Beerli P 2009 How to use migrate or why are Markov chain Monte Carlo programs difficult to use? In: Bertorelle G, Bruford MW, Hauffe HC, Rizzoli A, Vernesi C, eds. Population genetics for animal conservation, volume 17 of conservation biology. Cambridge: Cambridge University Press, 42–79.

[PLW002C9] Beerli P, Felsenstein J 2001 Maximum likelihood estimation of a migration matrix and effective population sizes in n subpopulations by using a coalescent approach. Proceedings of the National Academy of Sciences of the USA 98:4563–4568. 10.1073/pnas.081068098 11287657 PMC31874

[PLW002C10] Bekker RM, Kwak MM 2005 Life history traits as predictors of plant rarity, with particular reference to hemiparasitic Orobanchaceae. Folia Geobotanica 40:231–242. 10.1007/BF02803237

[PLW002C11] Bossolini E, Klahre U, Brandenburg A, Reinhardt D, Kuhlemeier C 2011 High resolution linkage maps of the model organism *Petunia* reveal substantial synteny decay with the related genome of tomato. Genome 54:327–340. 10.1139/g10-116 21491975

[PLW002C12] Brown JH 1984 On the relationship between abundance and distribution of species*.* The American Naturalist 124:255–279. 10.1086/284267

[PLW002C13] Chen S, Matsubara K, Omori T, Kokubun H, Kodama H, Watanabe H, Hashimoto G, Marchesi E, Bullrich L, Ando T 2007 Phylogenetic analysis of the genus *Petunia* (Solanaceae) based on the sequence of the *HF1* gene. Journal of Plant Research 120:385–397. 10.1007/s10265-006-0070-z 17353990

[PLW002C14] Choo J, Juenger TE, Simpson BB 2012 Consequences of frugivore-mediated seed dispersal for the spatial and genetic structures of a neotropical palm. Molecular Ecology 21:1019–1031. 10.1111/j.1365-294X.2011.05425.x 22229743

[PLW002C15] Cole CT 2003 Genetic variation in rare and common plants*.* Annual Review of Ecology, Evolution, and Systematics 34:213–237. 10.1146/annurev.ecolsys.34.030102.151717

[PLW002C16] Coppi A, Cecchi L, Mengoni A, Pustahija F, Tomović G, Selvi F 2014 Low genetic diversity and contrasting patterns of differentiation in the two monotypic genera *Halacsya* and *Paramoltkia* (Boraginaceae) endemic to the Balkan serpentines. Flora - Morphology, Distribution, Functional Ecology of Plants 209:5–14. 10.1016/j.flora.2013.11.002

[PLW002C17] Cornuet JM, Luikart G 1996 Description and power analysis of two tests for detecting recent population bottlenecks from allele frequency data. Genetics 144:2001–2014. 8978083 10.1093/genetics/144.4.2001 PMC1207747

[PLW002C18] Davies TJ, Smith GF, Bellstedt DU, Boatwright JS, Bytebier B, Cowling RM, Forest F, Harmon LJ, Muasya AM, Schrire BD, Steenkamp Y, Van der Bank M, Savolainen V 2011 Extinction risk and diversification are linked in a plant biodiversity hotspot. PLoS Biology 9:e1000620 10.1371/journal.pbio.1000620 21629678 PMC3101198

[PLW002C19] Derepas A, Dulieu H 1992 Inheritance of the capacity to transfer plastids by pollen parent in *Petunia hybrida* Hort. Journal of Heredity 83:6–10.

[PLW002C20] Dieringer D, Schlötterer C 2003 Microsatellite analyser (MSA): a platform independent analysis tool for large microsatellite data sets. Molecular Ecology Notes 3:167–169. 10.1046/j.1471-8286.2003.00351.x

[PLW002C21] Di Rienzo A, Peterson AC, Garza JC, Valdes AM, Slatkin M, Freimer NB 1994 Mutational processes of simple-sequence repeat loci in human populations. Proceedings of the National Academy of Sciences of the USA 91:3166–3170. 10.1073/pnas.91.8.3166 8159720 PMC43536

[PLW002C22] Di Rienzo A, Donnelly P, Toomajian C, Sisk B, Hill A, Petzl-Erler ML, Haines GK, Barch DH 1998 Heterogeneity of microsatellite mutations within and between loci, and implications for human demographic histories. Genetics 148:1269–1284. 9539441 10.1093/genetics/148.3.1269 PMC1460025

[PLW002C23] Drummond AJ, Rambaut A 2007 BEAST: Bayesian evolutionary analysis by sampling trees. BMC Evolutionary Biology 7:214 10.1186/1471-2148-7-214 17996036 PMC2247476

[PLW002C24] Drummond AJ, Rambaut A, Shapiro B, Pybus OG 2005 Bayesian coalescent inference of past population dynamics from molecular sequences. Molecular Biology and Evolution 22:1185–1192. 10.1093/molbev/msi103 15703244

[PLW002C25] Dun IS, Blattner FR 1987 Charons 36 to 40: multi enzyone, high capacity, recombination deficient replacement vectors with polylinkers and polystuffers. Nucleic Acids Research 15:2677–2698. 10.1093/nar/15.6.2677 3031608 PMC340677

[PLW002C26] Earl DA, Vonholdt BM 2012 Structure Harvester: a website and program for visualizing Structure output and implementing the Evanno method. Conservation Genetics Resources 4:359–361. 10.1007/s12686-011-9548-7

[PLW002C27] Edwards CE, Lindsay DL, Bailey P, Lance RF 2014 Patterns of genetic diversity in the rare *Erigeron lemmoni* and comparison with its more widespread congener, *Erigeron arisolius* (Asteraceae). Conservation Genetics 15:419–428. 10.1007/s10592-013-0549-9

[PLW002C28] Ellis JR, Pashley CH, Burke JM, Mccauley DEM 2006 High genetic diversity in a rare and endangered sunflower as compared to a common congener. Molecular Ecology 15:2345–2355. 10.1111/j.1365-294X.2006.02937.x 16842410

[PLW002C29] Ellstrand NC, Elam DR 1993 Population genetic consequences of small population size: implications for plant conservation. Annual Review of Ecology and Systematics 24:217–242. 10.1146/annurev.es.24.110193.001245

[PLW002C30] Evanno G, Regnaut S, Goudet J 2005 Detecting the number of clusters of individuals using the software STRUCTURE: a simulation study. Molecular Ecology 14:2611–2620. 10.1111/j.1365-294X.2005.02553.x 15969739

[PLW002C31] Excoffier L, Lischer HEL 2010 Arlequin suite ver 3.5: a new series of programs to perform population genetics analyses under Linux and Windows. Molecular Ecology Resources 10:564–567. 10.1111/j.1755-0998.2010.02847.x 21565059

[PLW002C32] Excoffier L, Smouse PE, Quattro JM 1992 Analysis of molecular variance inferred from metric distances among DNA haplotypes: application to human mitochondrial DNA restriction data. Genetics 131:479–491. 1644282 10.1093/genetics/131.2.479 PMC1205020

[PLW002C33] Falush D, Tephens MS, Pritchard JK 2003 Inference of population structure using multilocus genotype data: linked loci and correlated allele frequencies. Genetics 164:1567–1587. 12930761 10.1093/genetics/164.4.1567 PMC1462648

[PLW002C34] Felsenstein J 1985 Phylogenies and the comparative method. The American Naturalist 125:1–15. 10.1086/284325 31094602

[PLW002C35] Fu YX 1997 Statistical tests of neutrality of mutations against population growth, hitchhiking and background selection. Genetics 147:915–925. 9335623 10.1093/genetics/147.2.915 PMC1208208

[PLW002C36] Furches MS, Small RL, Furches A 2013 Genetic diversity in three endangered pitcher plant species (*Sarracenia*; Sarraceniaceae) is lower than widespread congeners. American Journal of Botany 100:2092–2101. 10.3732/ajb.1300037 24088341

[PLW002C37] Geyer CJ, Thompson EA 1995 Annealing Markov Chain Monte Carlo with applications to ancestral inference. Journal of the American Statistical Association 90:909–920. 10.1080/01621459.1995.10476590

[PLW002C38] Gibson JP, Rice SA, Stucke CM 2008 Comparison of population genetic diversity between a rare, narrowly distributed species and a common, widespread species of *Alnus* (Betulaceae). American Journal of Botany 95:588–596. 10.3732/ajb.2007316 21632385

[PLW002C39] Gitzendanner MA, Soltis PS 2000 Patterns of genetic variation in rare and widespread plant congeners. American Journal of Botany 87:783–792. 10.2307/2656886 10860909

[PLW002C40] Goudet J 1995 FSTAT version 1.2: a computer program to calculate F-statistics. Journal of Heredity 86:485–486.

[PLW002C41] Gübitz T, Hoballah ME, Dell'olivo A, Kuhlemeier C 2009 *Petunia* as a model system for the genetics and evolution of pollination syndromes. In: Gerats T, Strommer J, eds. Petunia evolutionary, developmental and physiological genetics. New York: Springer, 29–49.

[PLW002C42] Hamilton MB 1999 Four primer pairs for the amplification of chloroplast intergenic regions with intraspecific variation. Molecular Ecology 8:521–523. 10.1046/j.1365-294X.1999.00510.x 10199016

[PLW002C43] Hammer Ø, Harper DAT, Ryan PD 2001 PAST: paleontological statistics software package for education and data analysis. Palaeontologia Electronica 4:1–9.

[PLW002C44] Hamrick JL, Godt MJW 1989 Allozyme diversity in plant species. In: Brown AHD, Clegg MT, Kahler AL, Weir BS, eds. Plant population genetics, breeding and genetic resources. Sunderland: Sinauer, 43–63.

[PLW002C45] IUCN. 2012 The IUCN Red List of Threatened Species, version 3.1. International Union for Conservation of Nature and Natural Resources http://www.iucnredlist.org (6 January 2015).

[PLW002C46] Jakobsson M, Rosenberg NA 2007 CLUMPP: a cluster matching and permutation program for dealing with label switching and multimodality in analysis of population structure. Bioinformatics 23:1801–1806. 10.1093/bioinformatics/btm233 17485429

[PLW002C47] Jombart T 2008 ADEGENET: a R package for the multivariate analysis of genetic markers. Bioinformatics 24:1403–1405. 10.1093/bioinformatics/btn129 18397895

[PLW002C48] Jombart T, Devillard S, Balloux F 2010 Discriminant analysis of principal components: a new method for the analysis of genetically structured populations. BMC Genetics 11:94 10.1186/1471-2156-11-94 20950446 PMC2973851

[PLW002C49] Kalinowski ST 2005 HP-RARE 1.0: a computer program for performing rarefaction on measures of allelic richness. Molecular Ecology Notes 5:187–189. 10.1111/j.1471-8286.2004.00845.x

[PLW002C50] Kalinowski ST, Taper ML, Marshall TC 2007 Revising how the computer program CERVUS accommodates genotyping error increases success in paternity assignment. Molecular Ecology 16:1099–1106. 10.1111/j.1365-294X.2007.03089.x 17305863

[PLW002C51] Kettle CJ, Hollingsworth PM, Jaffré T, Moran B, Ennos RA 2007 Identifying the early genetic consequences of habitat degradation in a highly threatened tropical conifer, *Araucaria nemorosa* Laubenfels. Molecular Ecology 16:3581–3591. 10.1111/j.1365-294X.2007.03419.x 17845432

[PLW002C52] Klahre U, Gurba A, Hermann K, Saxenhofer M, Bossolini E, Guerin PM, Kuhlemeier C 2011 Pollinator choice in *Petunia* depends on two major genetic loci for floral scent production. Current Biology 21:730–739. 10.1016/j.cub.2011.03.059 21497087

[PLW002C53] Knapp S 2011 Rarity, species richness, and the threat of extinction—are plants the same as animals? PLoS Biology 9:e1001067 10.1371/journal.pbio.1001067 21629675 PMC3101195

[PLW002C54] Kokubun H, Nakano M, Tsukamoto T, Watanabe H, Hashimoto G, Marchesi E, Bullrich L, Basualdo IL, Kao TH, Ando T 2006 Distribution of self-compatible and self-incompatible populations of *Petunia axillaris* (Solanaceae) outside Uruguay. Journal of Plant Research 119:419–430. 10.1007/s10265-006-0002-y 16915365

[PLW002C55] Koopman MM, Carstens BC 2010 Conservation genetic inferences in the carnivorous pitcher plant *Sarracenia alata* (Sarraceniaceae). Conservation Genetics 11:2027–2038. 10.1007/s10592-010-0095-7

[PLW002C56] Kulcheski FR, Muschner VC, Lorenz-Lemke AP, Stehmann JR, Bonatto SL, Salzano FM, Freitas LB 2006 Molecular phylogenetic analysis of *Petunia* Juss. (Solanaceae). Genetica 126:3–14. 10.1007/s10709-005-1427-2 16502081

[PLW002C57] Lorenz-Lemke AP, Mäder G, Muschner VC, Stehmann JR, Bonatto SL, Salzano FM, Freitas LB 2006 Diversity and natural hybridization in a highly endemic species of *Petunia* (Solanaceae): a molecular and ecological analysis. Molecular Ecology 15:4487–4497. 10.1111/j.1365-294X.2006.03100.x 17107478

[PLW002C58] Lorenz-Lemke AP, Togni PD, Mäder G, Kriedt RA, Stehmann JR, Salzano FM, Bonatto SL, Freitas LB 2010 Diversification of plant species in a subtropical region of eastern South American highlands: a phylogeographic perspective on native *Petunia* (Solanaceae). Molecular Ecology 19:5240–5251. 10.1111/j.1365-294X.2010.04871.x 21040052

[PLW002C59] Mantel N 1967 The detection of disease clustering and a generalized regression approach. Cancer Research 27:209–220. 6018555

[PLW002C60] Markham J 2014 Rare species occupy uncommon niches. Scientific Reports 4:6012 10.1038/srep06012 25110113 PMC5381397

[PLW002C61] Marshall TC, Slate J, Kruuk LEB, Pemberton JM 1998 Statistical confidence for likelihood-based paternity inference in natural populations. Molecular Ecology 7:639–655. 10.1046/j.1365-294x.1998.00374.x 9633105

[PLW002C62] Miller MP 2005 Alleles In Space: computer software for the joint analysis of interindividual spatial and genetic information. Journal of Heredity 96:722–724. 10.1093/jhered/esi119 16251514

[PLW002C63] Nicholas KB, Nicholas KBJ 1997 GeneDoc: a tool for editing and annotating multiple sequences alignments. http://iubio.bio.indiana.edu/soft/molbio/ibmpc/genedoc-readme.html (6 January 2015).

[PLW002C64] Oleas NH, Von Wettber EJB, Negrón-Ortiz V 2014 Population genetics of the Federally Threatened Miccosukee gooseberry (*Ribes echinellum*), an endemic North American species. Conservation Genetics 15:749–755.

[PLW002C65] Pérez de Paz J, Caujapé-Castells J 2013 A review of the allozyme data set for the Canarian endemic flora: causes of the high genetic diversity levels and implications for conservation. Annals of Botany 111:1059–1073. 10.1093/aob/mct076 23609020 PMC3662517

[PLW002C67] Phillips RD, Peakall R, Hutchinson HF, Linde CC, Xu T, Dixon KW, Hopper SD 2014 Specialized ecological interactions and plant species rarity: the role of pollinators and mycorrhizal fungi across multiple spatial scales. Biological Conservation 169:285–295. 10.1016/j.biocon.2013.11.027

[PLW002C68] Piry S, Luikart G, Cornuet J-M 1999 BOTTLENECK: a computer program for detecting recent reductions in the effective population size using allele frequency data. Journal of Heredity 90:502–503. 10.1093/jhered/90.4.502

[PLW002C69] Premoli AC, Souto CP, Allnutt TR, Newton AC 2001 Effects of population disjunction on isozyme variation in the widespread *Pilgerodendron uviferum*. Heredity 87:337–343. 10.1046/j.1365-2540.2001.00906.x 11737280

[PLW002C70] Pritchard JK, Stephens M, Donnelly P 2000 Inference of population structure using multilocus genotype data. Genetics 155:945–959. 10835412 10.1093/genetics/155.2.945 PMC1461096

[PLW002C97] Rabinowitz D 1981 Seven forms of rarity. In: Synge H, ed. The biological aspects of rare plant conservation. Chichester: John wiley, 205–217.

[PLW002C71] Rambaut A 2008 FigTree, version 1.4: tree figure drawing tool. http://tree.bio.ed.ac.uk/software/figtree/ (6 January 2015).

[PLW002C72] Rambaut A, Suchard MA, Xie D, Drummond AJ 2013 Tracer, version 1.5. http://beast.bio.ed.ac.uk/Tracer (6 January 2015).

[PLW002C73] Rannala B, Yang ZH 1996 Probability distribution of molecular evolutionary trees: a new method of phylogenetic inference. Journal of Molecular Evolution 43:304–311. 10.1007/BF02338839 8703097

[PLW002C74] Raymond M, Rousset F 1995 GENEPOP (version 1.2): population genetics software for exact tests and ecumenicism. Journal of Heredity 86:248–249.

[PLW002C75] R Development Core Team. 2011 R: a language and environment for statistical computing. http://www.r-project.org/ (6 January 2015).

[PLW002C76] Reck-Kortmann M, Silva-Arias GA, Segatto ALA, Mäder G, Bonatto SL, De Freitas LB 2014 Multilocus phylogeny reconstruction: new insights into the evolutionary history of the genus *Petunia*. Molecular Phylogenetics and Evolution 81:19–28. 10.1016/j.ympev.2014.08.022 25196589

[PLW002C77] Rosenberg NA 2004 DISTRUCT: a program for the graphical display of population structure. Molecular Ecology Notes 4:137–138. 10.1046/j.1471-8286.2003.00566.x

[PLW002C78] Roy A, Frascaria N, Mackay J, Bousquet J 1992 Segregating random ampliﬁed polymorphic DNAs (RAPDs) in *Betula alleghaniensis*. Theoretical and Applied Genetics 85:173–180. 24197301 10.1007/BF00222856

[PLW002C79] Saitou N, Nei N 1987 The neighbor-joining method: a new method for reconstructing phylogenetic trees. Molecular Biology and Evolution 4:406–425. 3447015 10.1093/oxfordjournals.molbev.a040454

[PLW002C80] Sang T, Crawford DJ, Stuessy TF 1997 Chloroplast DNA phylogeny, reticulate evolution, and biogeography of *Paeonia* (Paeoniaceae). American Journal of Botany 84:1120–1136. 10.2307/2446155 21708667

[PLW002C95] Schwartz MW, Simberloff D 2001 Taxon size predicts rates of rarity in vascular plants. Ecology Letters 4:464–469.

[PLW002C81] Segatto ALA, Cazé ALR, Turchetto C, Klahre U, Kuhlemeier C, Bonatto SL, Freitas LB 2014a Nuclear and plastid markers reveal the persistence of genetic identity: a new perspective on the evolutionary history of *Petunia exserta*. Molecular Phylogenetics and Evolution 70:504–512. 10.1016/j.ympev.2013.10.011 24161675

[PLW002C82] Segatto ALA, Ramos-Fregonezi AMC, Bonatto SL, Freitas LB 2014b Molecular insights into the purple-flowered ancestor of garden petunias. American Journal of Botany 101:119–127. 10.3732/ajb.1300223 24368755

[PLW002C83] Sheth SN, Angert AL 2014 The evolution of environmental tolerance and range size: a comparison of geographically restricted and widespread *Mimulus*. Evolution 68:2917–2931. 10.1111/evo.12494 25066881

[PLW002C84] Slatyer RA, Hirst M, Sexton JP 2013 Niche breadth predicts geographical range size: a general ecological pattern. Ecology Letters 16:1104–1114. 10.1111/ele.12140 23773417

[PLW002C85] Stehmann JR, Lorenz-Lemke AP, Freitas LB, Semir J 2009 The genus *Petunia*. In: Gerats T, Strommer J, eds. Petunia evolutionary, developmental and physiological genetics. New York: Springer, 1–28.

[PLW002C96] Stehmann JR, Semir J 2005 New species of *Calibrachoa* and *Petunia* (Solanaceae) from subtropical South America. Systematic Botany Monographs 104:341–348.

[PLW002C86] Tajima F 1989 Statistical-method for testing the neutral mutation hypothesis by DNA polymorphism. Genetics 123:585–595. 2513255 10.1093/genetics/123.3.585 PMC1203831

[PLW002C87] Tamura K, Stecher G, Peterson D, Filipski A, Kumar S 2013 MEGA6: molecular evolutionary genetics analysis. Version 6.0. Molecular Biology and Evolution 30:2725–2729. 10.1093/molbev/mst197 24132122 PMC3840312

[PLW002C88] Turchetto C, Fagundes NJR, Segatto ALA, Kuhlemeier C, Solís Neffa VG, Speranza PR, Bonatto SL, Freitas LB 2014a Diversification in the South American Pampas: the genetic and morphological variation of the widespread *Petunia axillaris* complex (Solanaceae). Molecular Ecology 23:374–389. 10.1111/mec.12632 24372681

[PLW002C89] Turchetto C, Segatto ALA, Telles MPC, Diniz-Filho JAF, Freitas LB 2014b Infraspecific classification reflects genetic differentiation in the widespread *Petunia axillaris* complex: a comparison among morphological, ecological, and genetic patterns of geographic variation. Perspectives in Plant Ecology, Evolution and Systematics 16:75–82. 10.1016/j.ppees.2014.01.002

[PLW002C90] Turchetto C, Lima JS, Rodrigues DM, Bonatto SL, Freitas LB 2015a Pollen dispersal and breeding structure in a hawkmoth-pollinated Pampa grasslands species *Petunia axillaris* (Solanaceae). Annals of Botany 115:939–948. 10.1093/aob/mcv025 25808656 PMC4407064

[PLW002C91] Turchetto C, Segatto ALA, Beduschi J, Bonatto SL, Freitas LB 2015b Genetic differentiation and hybrid identification using microsatellite markers in closely related wild species. AoB PLANTS 7: plv084; 10.1093/aobpla/plv084 PMC4565426 26187606

[PLW002C92] Venail J, Dell'olivo A, Kuhlemeier C 2010 Speciation genes in the genus *Petunia*. Philosophical Transactions of the Royal Society B: Biological Sciences 365:461–468. 10.1098/rstb.2009.0242 PMC2838266 20047872

[PLW002C93] Wang ZF, Hamrick JL, Godt MJW 2004 High genetic diversity in *Sarracenia leucophylla* (Sarraceniaceae), a carnivorous wetland herb*.* Journal of Heredity 95:234–243. 10.1093/jhered/esh043 15220390

[PLW002C94] Watanabe H, Ando T, Tsukamoto T, Hashimoto G, Marchesi E 2001 Cross-compatibility of *Petunia exserta* with other petunia taxa. Journal of the Japanese Society for Horticultural Science 70:33–40. 10.2503/jjshs.70.33

