# Supplementary material for: High levels of genetic diversity and population structure in an endemic and rare species: implications for conservation
Source: AoB Plants. 2016 Jan 14;8:plw002. doi: 10.1093/aobpla/plw002 (PMC4768524; doi:10.1093/aobpla/plw002)
Supplement: Additional Information [file supp_8_plw002_index.html]

High levels of genetic diversity and population structure in an endemic and rare species: implications for conservation — Additional Information 

# High levels of genetic diversity and population structure in an endemic and rare species: implications for conservation

## Additional Information

Additional Information

- Supplementary Figure 1 - docx file
- Supplementary Table 1 - docx file
- Supplementary Table 2 - xlsx file
- Supplementary Table 3 - docx file
- Supplementary Table 4 - docx file
- Supplementary Table 5 - docx file
- Supplementary Table 6 - docx file
- Supplementary Table 7 - docx file
