# Supplementary material for: High levels of genetic diversity and population structure in an endemic and rare species: implications for conservation
Source: AoB Plants. 2016 Jan 14;8:plw002. doi: 10.1093/aobpla/plw002 (PMC4768524; doi:10.1093/aobpla/plw002)

**Figure S1.** Evolutionary relationships among plastid haplotypes of *P. secreta* and congeners from Serra do Sudeste according. Bayesian Inference with clade posterior probability (> 0.5) indicated to selected branches.


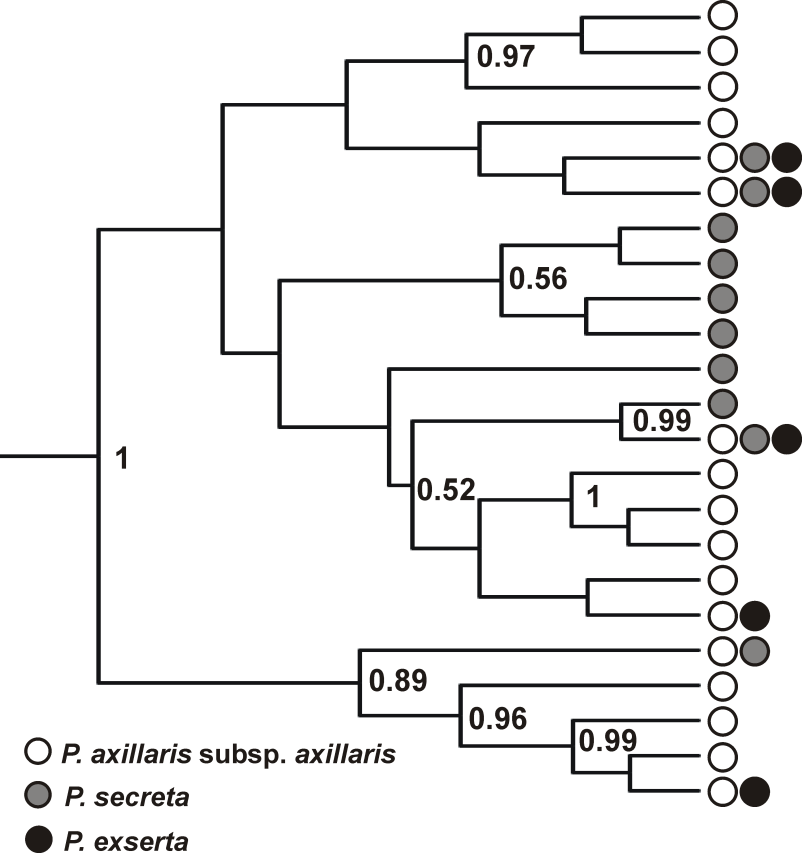

Supplement: Additional Information [file supp_plw002_plw002supp_fig1.docx]
