# Supplementary material for: High levels of genetic diversity and population structure in an endemic and rare species: implications for conservation
Source: AoB Plants. 2016 Jan 14;8:plw002. doi: 10.1093/aobpla/plw002 (PMC4768524; doi:10.1093/aobpla/plw002)
Supplement: Additional Information [file supp_plw002_plw002supp_table1.docx]

**Table S1.** GenBank accession numbers of plastid sequences.

| ***Sample*** | ***trnH-psbA*** | ***trnS-trnG*** | ***Sample*** | ***trnH-psbA*** | ***trnS-trnG*** |
| --- | --- | --- | --- | --- | --- |
| P. sec 1 | AY772895 | KM260457 | P. sec 34 | KM260440 | KM260484 |
| P. sec 2 | AY772896 | KM260458 | P. sec 35 | KM260441 | KM260485 |
| P. sec 3 | KM260415 | KM260459 | P. sec 36 | KM260442 | KM260486 |
| P. sec 4 | AY772897 | KC832915 | P. sec 37 | KM260443 | KM260487 |
| P. sec 5 | KM260416 | KM260460 | P. sec 38 | KM260444 | KM260488 |
| P. sec 6 | KJ801277 | KJ801293 | P. sec 39 | KM260445 | KM260489 |
| P. sec 7 | KM260417 | KM260461 | P. sec 40 | KM260446 | KM260490 |
| P. sec 8 | KM260418 | KM260462 | P. sec 41 | KM260447 | KM260491 |
| P. sec 9 | KM260419 | KM260463 | P. sec 42 | KM260448 | KM260492 |
| P. sec 10 | KM260420 | KM260464 | P. sec 43 | KM260449 | KM260493 |
| P. sec 11 | KM260421 | KM260465 | P. sec 44 | KM260450 | KM260494 |
| P. sec 12 | KJ801273 | KJ801289 | P. sec 45 | KM260451 | KM260495 |
| P. sec 13 | KJ801274 | KJ801290 | P. sec 46 | KM260452 | KM260496 |
| P. sec 14 | KJ801275 | KJ801291 | P. sec 47 | KM260453 | KM260497 |
| P. sec 15 | KM260422 | KM260466 | P. sec 48 | KM260454 | KM260498 |
| P. sec 16 | KM260423 | KM260467 | P. sec 49 | KM260455 | KM260499 |
| P. sec 17 | KJ801276 | KJ801292 | P. sec 50 | KM260456 | KM260500 |
| P. sec 18 | KM260424 | KM260468 | P. sec 140 | KR010666 | KR010681 |
| P. sec 19 | KM260425 | KM260469 | P. sec 141 | KR010667 | KR010682 |
| P. sec 20 | KM260426 | KM260470 | P. sec 145 | KR010668 | KR010683 |
| P. sec 21 | KM260427 | KM260471 | P. sec 162 | KR010669 | KR010684 |
| P. sec 22 | KM260428 | KM260472 | P. sec 164 | KR010670 | KR010685 |
| P. sec 23 | KM260429 | KM260473 | P. sec 165 | KR010671 | KR010686 |
| P. sec 24 | KM260430 | KM260474 | P. sec 429 | KR010672 | KR010687 |
| P. sec 25 | KM260431 | KM260475 | P. sec 432 | KR010673 | KR010688 |
| P. sec 26 | KM260432 | KM260476 | P. sec 434 | KR010674 | KR010689 |
| P. sec 27 | KM260433 | KM260477 | P. sec 569 | KR010675 | KR010690 |
| P. sec 28 | KM260434 | KM260478 | P. sec 580 | KR010676 | KR010691 |
| P. sec 29 | KM260435 | KM260479 | P. sec 581 | KR010677 | KR010692 |
| P. sec 30 | KM260436 | KM260480 | P. sec 756 | KR010678 | KR010693 |
| P. sec 31 | KM260437 | KM260481 | P. sec 757 | KR010679 | KR010694 |
| P. sec 32 | KM260438 | KM260482 | P. sec 760 | KR010680 | KR010695 |
| P. sec 33 | KM260439 | KM260483 |  |  |  |
