# Supplementary material for: High levels of genetic diversity and population structure in an endemic and rare species: implications for conservation
Source: AoB Plants. 2016 Jan 14;8:plw002. doi: 10.1093/aobpla/plw002 (PMC4768524; doi:10.1093/aobpla/plw002)
Supplement: Additional Information [file supp_plw002_plw002supp_table3.docx]

**Table S3.** Genetic distance based on microsatellite shared alleles among *Petunia secreta* collection sites.

|  | **Pop1** | **Pop2** | **Pop3** | **Pop4** | **Pop5** | **Pop6** | **Pop7** | **Pop8** | **Pop9** | **Pop10** | **Pop11** | **Pop12** |
| --- | --- | --- | --- | --- | --- | --- | --- | --- | --- | --- | --- | --- |
| **Pop2** | 0.53 |  |  |  |  |  |  |  |  |  |  |  |
| **Pop3** | 0.72 | 0.63 |  |  |  |  |  |  |  |  |  |  |
| **Pop4** | 0.57 | 0.70 | 0.40 |  |  |  |  |  |  |  |  |  |
| **Pop5** | 0.58 | 0.70 | 0.53 | 0.40 |  |  |  |  |  |  |  |  |
| **Pop6** | 0.70 | 0.70 | 0.37 | 0.40 | 0.63 |  |  |  |  |  |  |  |
| **Pop7** | 0.52 | 0.70 | 0.57 | 0.44 | 0.55 | 0.54 |  |  |  |  |  |  |
| **Pop8** | 0.82 | 0.82 | 0.64 | 0.57 | 0.79 | 0.57 | 0.69 |  |  |  |  |  |
| **Pop9** | 0.60 | 0.83 | 0.67 | 0.47 | 0.50 | 0.70 | 0.51 | 0.71 |  |  |  |  |
| **Pop10** | 0.65 | 0.81 | 0.77 | 0.54 | 0.62 | 0.69 | 0.63 | 0.62 | 0.54 |  |  |  |
| **Pop11** | 0.57 | 0.68 | 0.64 | 0.32 | 0.36 | 0.54 | 0.55 | 0.73 | 0.61 | 0.58 |  |  |
| **Pop12** | 0.56 | 0.75 | 0.56 | 0.50 | 0.56 | 0.56 | 0.27 | 0.69 | 0.53 | 0.65 | 0.59 |  |
| **Pop13** | 0.66 | 0.65 | 0.63 | 0.68 | 0.65 | 0.60 | 0.61 | 0.79 | 0.76 | 0.73 | 0.62 | 0.65 |
