# Supplementary material for: High levels of genetic diversity and population structure in an endemic and rare species: implications for conservation
Source: AoB Plants. 2016 Jan 14;8:plw002. doi: 10.1093/aobpla/plw002 (PMC4768524; doi:10.1093/aobpla/plw002)
Supplement: Additional Information [file supp_plw002_plw002supp_table4.docx]

**Table S4.** *Petunia secreta* measures (mm) of morphological floral traits.

| **Collection Site** | **Flower** | **Corolla tube length** | **Distance between the anthers** | **Corolla**  **diameter** | **Total flower**  **size** |
| --- | --- | --- | --- | --- | --- |
| Pedra do Segredo | 1 | 44.40 | 1.90 | 43.60 | 65.60 |
|  | 2 | 45.30 | 1.70 | 42.60 | 66.60 |
|  | 3 | 40.70 | 1.10 | 43.50 | 62.30 |
| Partial Average |  | 43.47 | 1.57 | 43.27 | 64.83 |
| BR290 | 1 | 47.00 | 1.41 | 40.65 | 66.21 |
|  | 2 | 47.20 | 1.84 | 40.57 | 68.59 |
|  | 3 | 40.21 | 3.01 | 34.68 | 58.05 |
|  | 4 | 37.69 | 2.65 | 32.13 | 53.16 |
|  | 5 | 43.42 | 1.02 | 42.38 | 65.93 |
|  | 6 | 44.20 | 1.49 | 40.63 | 61.28 |
|  | 7 | 46.30 | 5.57 | 45.97 | 69.35 |
|  | 8 | 46.22 | 5.90 | 45.81 | 66.50 |
|  | 9 | 45.44 | 5.81 | 50.99 | 68.34 |
|  | 10 | 45.93 | 1.70 | 44.48 | 65.76 |
|  | 11 | 45.01 | 4.83 | 35.83 | 62.18 |
|  | 12 | 40.99 | 1.73 | 33.44 | 56.23 |
| Partial Average |  | 44.13 | 3.08 | 40.63 | 63.47 |
| Average |  | 43.80 | 2.33 | 41.95 | 64.15 |
