# Supplementary material for: High levels of genetic diversity and population structure in an endemic and rare species: implications for conservation
Source: AoB Plants. 2016 Jan 14;8:plw002. doi: 10.1093/aobpla/plw002 (PMC4768524; doi:10.1093/aobpla/plw002)
Supplement: Additional Information [file supp_plw002_plw002supp_table5.docx]

**Table S5.** Plastid haplotypes per individual per collection site.

| **Site** | **Individuals** | **Haplotype** |
| --- | --- | --- |
| Pop1 | P.sec 1, P.sec 2 | H1 |
| Pop2 | P.sec 3 | H2 |
| Pop3 | P.sec 4 | H3 |
| Pop4 | P.sec 5 | H2 |
| Pop5 | P.sec 6 | H4 |
| Pop6 | P.sec 7 | H2 |
| Pop7 | P.sec 33, P.sec 35 | H6 |
|  | P.sec 39 | H7 |
|  | P.sec 8-18, P.sec 24-33, P.sec 34, P.sec 36-38 | H1 |
| Pop8 | P.sec 19 | H5 |
| Pop9 | P.sec 20, P.sec 21 | H2 |
| Pop10 | P.sec 22 | H2 |
| Pop11 | P.sec 23 | H2 |
| Pop12 | P.sec 40-46, P.sec 48, P.sec 50 | H1 |
|  | P.sec 47. P.sec 49 | H8 |
| Pop13 | P.sec 140, P.sec 141, P.sec 145, P.sec 165, P.sec 434, P.sec 580 | H9 |
|  | P.sec 162, P.sec 164, P.sec 429, P.sec 432, P.sec 569, P.sec 581, P.sec 756, P.sec 757, P.sec 760 | H2 |
