# Supplementary material for: High levels of genetic diversity and population structure in an endemic and rare species: implications for conservation
Source: AoB Plants. 2016 Jan 14;8:plw002. doi: 10.1093/aobpla/plw002 (PMC4768524; doi:10.1093/aobpla/plw002)
Supplement: Additional Information [file supp_plw002_plw002supp_table6.docx]

**Table S6.** Genetic distance based on shared microsatellite alleles among *Petunia* species from Serra do Sudeste.

|  | ***P. axillaris*** | ***P. exserta*** |
| --- | --- | --- |
| ***P. exserta*** | 0.42 |  |
| ***P. secreta*** | 0.41 | 0.50 |

Note: *P. axillaris* and *P. exserta* data were obtained from Turchetto et al. (2015).
