# Supplementary material for: High levels of genetic diversity and population structure in an endemic and rare species: implications for conservation
Source: AoB Plants. 2016 Jan 14;8:plw002. doi: 10.1093/aobpla/plw002 (PMC4768524; doi:10.1093/aobpla/plw002)
Supplement: Additional Information [file supp_plw002_plw002supp_table7.docx]

**Table S7.** Genetic distance based on shared microsatellite alleles among *Petunia* species separating *Petunia secreta* into two groups according to the geographic distribution in the Pedra do Segredo locality (I; Pop1-12) and BR 290 collection site (II; Pop13) and clustering analyses.

|  | ***P. axillaris*** | ***P. exserta*** | ***P. secreta*  I** |
| --- | --- | --- | --- |
| ***P. exserta*** | 0.42 |  |  |
| ***P. secreta* I** | 0.46 | 0.53 |  |
| ***P. secreta* II** | 0.55 | 0.63 | 0.62 |

Note: *P. axillaris* and *P. exserta* were obtained from Turchetto et al. (2015).
